# Magneto-oncology: a radical pair primer

**DOI:** 10.3389/fonc.2025.1539718

**Published:** 2025-03-07

**Authors:** P. J. Hore

**Affiliations:** Department of Chemistry, Oxford University, Oxford, United Kingdom

**Keywords:** radical pair mechanism (RPM), spin chemistry, electron spin, magnetobiology, magnetic field effects (MFE)

## Abstract

There are few well-established biophysical mechanisms by which external magnetic fields can influence the biochemistry of molecules in living systems. The radical pair mechanism is arguably the most promising. In this mini-review I summarize the characteristics of radical pairs in a way that may be useful to those engaged in the field of magneto-oncology. The intention is to help researchers decide whether an observed biomedical magnetic field effect could have its origin in radical pair biochemistry. Armed with a physically plausible interaction mechanism, it may be possible to devise and refine a theoretical model and thereby iteratively optimise therapeutic protocols. Such an approach may also help identify experimental artefacts

## Introduction

Magnetic nanoparticles, guided to specific locations by external magnetic fields, have a variety of applications in cancer treatment. They can deliver drugs or genetic material, produce localized heating (magnetic hyperthermia), enhance immune cell activation, and help visualise tumours (magnetic resonance imaging and magnetic particle imaging). The magnetic fields involved are typically stronger than 10 millitesla and the fundamental physics – magnetic forces, radiofrequency heating, spin relaxation, and so on – is well understood. By contrast, the primary interaction mechanisms behind strategies that do not involve nanoparticles are often obscure. The problem is that the energy with which even a 1 tesla magnetic field interacts with a single molecule is a great deal smaller than the energy of the naturally occurring random fluctuations in atomic positions and molecular orientations (1). Even for paramagnetic molecules like free radicals, we can expect that any magnetic field effect on the rates or yields of (bio)chemical reactions should be overwhelmed by thermal noise. No matter how efficient any subsequent amplification mechanisms may be, there can be no magnetic field effect if the primary signal-to-noise ratio is less than one (2).

However, a well-established interaction mechanism does exist for which this thermodynamic argument is irrelevant: the radical pair mechanism (3–10). Over the last fifty years, it has been used, often quantitatively, to account for hundreds of laboratory studies of magnetic field effects on free radical reactions. Although convincing examples in biology have been scarce, there is no reason why the mechanism could not operate in living systems (11), for instance in the magnetic compass of migratory birds (12–17).

My purpose in writing this article is to summarize the characteristics of the radical pair mechanism in a way that might be useful to those working in the field of magneto-oncology. The intention is to help researchers decide whether an observed biomedical magnetic field effect could have its origin in radical pair biochemistry. Armed with a physically plausible interaction mechanism, it may be possible to devise and refine a theoretical model and thereby iteratively optimise therapeutic protocols. It may also help avoid experimental artefacts, [of which magnetobiology (18–30) seems to have more than its fair share (11)] and allow *in silico* investigation of features of the interaction mechanism that do not readily lend themselves to experimental study. In the course of this primer, little attempt will be made to explain the spin physics underlying the radical pair mechanism – that can be found elsewhere (3, 9, 31, 32) and in some of the articles cited below. The text starts with a brief summary of the mechanism itself, continues with descriptions of its various manifestations, and ends with a few concluding remarks. Some basic quantitative aspects of the mechanism are summarized in the **Supplementary Material**.

## Radical pair mechanism

**Figure 1:**
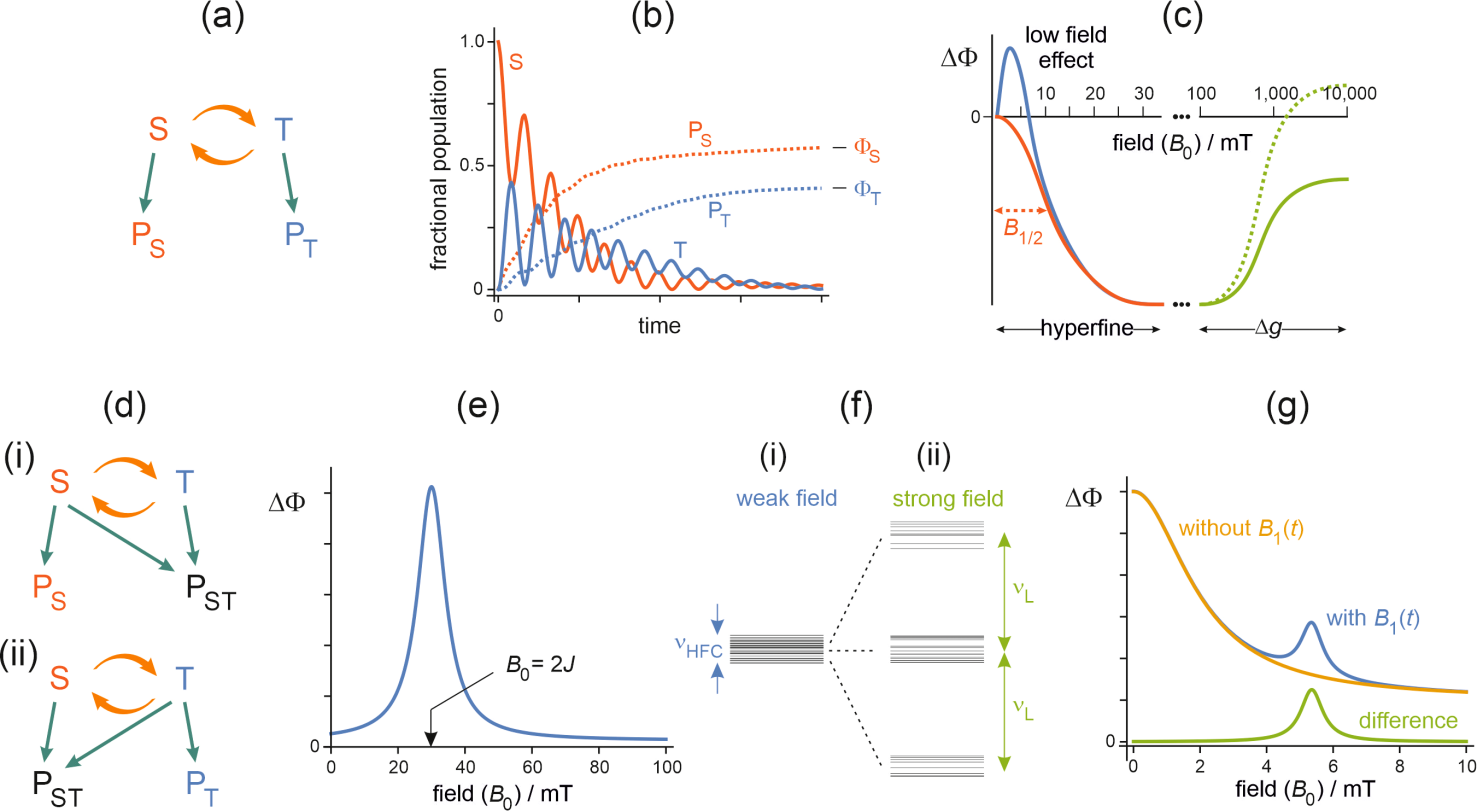
**(a)** The simplest radical pair reaction scheme. S and T are the singlet and triplet forms of the radical pair. The orange arrows represent the oscillatory interconversion of S and T by hyperfine and Zeeman interactions. S and T react spin-selectively to form singlet and triplet products, P_S_ and P_T_, respectively. **(b)** Schematic time-dependence of radical-pair and product states. Starting in the singlet state, S and T interconvert coherently while reacting to give P_S_ and P_T_, as in scheme **(a)**. The quantum beat frequencies are typically in the megahertz to gigahertz range depending on the spin interactions of the two electrons. The populations of the products build up to their final levels, $\Phi_{\text{S}}$ and $\Phi_{\text{T}}$ , where $\Phi_{\text{S}}+\Phi_{\text{T}}=1$ . **(c)** Schematic changes in the yield of a reaction product, $\Delta \Phi =\Phi (B_{0})-\Phi (0)$ , induced by a static external magnetic field of strength $B_{0}$ . Using the notation X→Y , where X is the initial spin state of the radical pair and Y is the reaction product, this figure is appropriate for $\text{S}\to {\text{P}}_{\text{T}}$ [reaction schemes **(a)** and **(d)** (ii)] and $\text{T}\to {\text{P}}_{\text{S}}$ [**(a)** and **(d)** (i)]. The sign of ΔΦ is inverted for $\text{S}\to {\text{P}}_{\text{S}}$ [**(a)** and **(d)** (i)] and $\text{T}\to {\text{P}}_{\text{T}}$ [**(a)** and **(d)** (ii)]. The values of $B_{0}$ on the horizontal axis give an impression of the magnetic fields at which the various features normally occur for organic radicals with effective hyperfine interactions ≈ 1 mT and Δ*g* ≈ 0.001. The left-hand side shows the behaviour expected for radical pairs with short-lived (red) and long-lived (blue) spin coherence. For the red line, $B_{1/2}$ is the magnetic field at which ΔΦ equals half its value at the plateau (in this case when $B_{0}$ ≈ 30 mT). The right-hand side shows two possible effects of the Δ*g* mechanism (green). Effects of exchange and dipolar interactions have been ignored. Δ*g* is defined in the **Supplementary Material**. **(d)** Alternative radical-pair reaction schemes. (i) S reacts spin-selectively to form P_S_ while S and T react non-selectively to give a product P_ST_. (ii) T reacts spin-selectively to form P_T_ while S and T react non-selectively to give a product P_ST_. **(e)** Schematic field-dependence of the reaction yield for a radical pair with a strong exchange interaction. Using the notation in **(c)**, this figure is appropriate for $\text{S}\to {\text{P}}_{\text{T}}$ [reaction schemes **(a)** and **(d)**(ii)] and $\text{T}\to {\text{P}}_{\text{S}}$ [**(a)** and **(d)**(i)]. The extremum occurs when $B_{0}=2J$ where *J* is the strength of the exchange interaction. **(f)** Schematic spin energy-levels of a radical pair in a static magnetic field (i) weaker and (ii) stronger than the hyperfine, exchange and dipolar interactions. $\nu_{\text{HFC}}$ is the maximum resonance frequency in a weak static field, determined mainly by hyperfine interactions. $\nu_{\text{L}}$ is the Larmor frequency, determined by the strong Zeeman interaction. **(g)** Schematic *B*_0_-dependence of the reaction yield with and without a weak 150 MHz time-dependent field $B_{1}$ . The resonance appears when $B_{0}$ = 150/28 = 5.4 mT.

Radicals are molecules that contain an unpaired electron whose spin angular momentum (or simply, spin) is associated with a magnetic moment (33–35). In simple terms, the origin of magnetic field effects on pairs of radicals can be understood by reference to **Figure 1a**. The two unpaired electrons, one in each radical, can be in either singlet (S) or triplet (T) states depending, roughly speaking, on whether their spins are mutually aligned antiparallel (↑↓) or parallel (↑↑), respectively. The reactivity of this pair of radicals is subject to spin-selection rules: S-pairs are formed from S-state reactants, and react to give S-state products, and similarly for T-pairs (**Figure 1a**). Following spin-selective formation, the S and T states interconvert coherently, typically at megahertz or gigahertz frequencies depending on the spin interactions of the two electrons (**Figure 1b**) (14). An external static, radiofrequency, or microwave magnetic field acting on the two electrons (Zeeman interactions) can alter the extent and timing of S↔T interconversion and hence the probabilities that the pair reacts to form the singlet (P_S_) or the triplet (P_T_) product (**Figures 1a, b**). Competition between the two pathways means that an increase in the final yield ( $\Phi_{\text{S}}$ or $\Phi_{\text{T}}$ ) of one product is matched by a decrease in that of the other. In reality, the pattern of S↔T “quantum beats” is considerably more complex than shown in **Figure 1b** because each electron spin has (hyperfine) interactions with the spins of several atomic nuclei (e.g. ^1^H and ^14^N) (36–38).

All the relevant magnetic interactions of the two electrons – with external magnetic fields (Zeeman), with nuclear spins (hyperfine), and with each other (exchange and dipolar) – are normally orders of magnitude smaller than the thermal energy, *k*_B_ *T* (Boltzmann’s constant times temperature, equivalent to 2.6 kJ mol^−1^ at physiological temperature). An applied magnetic field cannot therefore break chemical bonds or otherwise initiate new (bio)chemical transformations. It can only modify the yields of existing reactions. Uniquely, this is possible for radical pairs because the electron spins couple so weakly to their surroundings. The non-equilibrium spin states created by spin-selective reactions can persist for times as long as a microsecond before the coherences vanish and the S:T ratio reaches its equilibrium value of 1:3. A spin relaxation time of ~1 μs gives enough time for an external magnetic field stronger than ~100 µT to have a significant effect on the quantum beats and therefore the reaction yields (39, 40). Radical pairs with lifetimes in excess of 1 μs show very weak sensitivity to magnetic fields because the spin correlation decays before any products can be formed. Magnetic fields weaker than ~100 µT would require improbably slow spin relaxation and are therefore highly unlikely to produce significant effects *in vivo* unless there has been evolutionary pressure to optimise the sensitivity.

More detailed introductory material can be found in ref (14). which aims to “explain the chemical and physical aspects of radical-pair magnetoreception to biologists and the biological and chemical aspects to physicists”.

## Static magnetic field effects

The most common manifestation of the radical pair mechanism is the dependence of the yields of the reaction products on the strength (magnetic flux density) of a static external magnetic field (**Figure 1c**). Whether the yield is increased or decreased depends on the properties of the radicals, the strength of the field, the initial spin state (S or T), and which product (P_S_ or P_T_) one looks at.

The shape of **Figure 1c** (appropriate for the reaction scheme in **Figure 1a** and the slightly more complicated ones in **Figure 1d**) has three origins. In magnetic fields up to about 100 mT, S↔T interconversion is dominated by the hyperfine interactions, and the reaction yields usually have a sigmoidal field-dependence (41–44). The “half-field” parameter, *B*_1/2_, is normally comparable to the effective hyperfine interaction of the two radicals (defined in the **Supplementary Material**), typically a few millitesla for organic radicals (41, 45). Larger values of *B*_1/2_ are expected if one or both radicals undergo rapid spin relaxation (46, 47). If the radicals are long-lived and spin-relax sufficiently slowly, the reaction yields may have an extremum known as the low field effect (31) which boosts the sensitivity to magnetic fields weaker than *B*_1/2_ and flips the sign of the effect. For stronger fields, S↔T interconversion is driven by the difference in the Zeeman interactions of the two electrons with the applied magnetic field (Δ*g* mechanism) (48, 49). Finally (not shown in **Figure 1c**), the rate at which spin coherence decays is sometimes field-dependent such that strong magnetic fields give rise to further changes in reaction yields (50).

If the radicals are not randomly oriented, the effect of a static magnetic field may depend on its direction (51–53). This is thought to be the basis of the magnetic compass sense of migratory songbirds (12–14, 39). Exact reversal of the field direction should have no effect on the spin dynamics whether the radicals are oriented or not (54, 55).

The field-dependence of the reaction yields is profoundly different when the exchange or dipolar interaction of the two electrons is larger than the hyperfine couplings. Strong interactions suppress the effects of weak magnetic fields and give rise to a “2*J* resonance” when the Zeeman interaction matches the exchange coupling (**Figure 1e**) (3, 56). Exchange interactions are generally negligible for radicals separated by more than 1.0 to 1.5 nm; dipolar interactions have a longer range: ~1 mT at 1.4 nm; ~0.1 mT at 3.0 nm (57).

## Low-frequency magnetic field effects (< 1 MHz)

Radical pairs that show static magnetic field effects rarely have lifetimes longer than 1 μs. This means that oscillating magnetic fields with frequencies much below 1 MHz are effectively static as far as the radical pairs are concerned (58, 59). As there is unlikely to be any correlation between the phase of an external alternating field and the instant at which radical pairs are formed, any observed effect will be an average over a period of the time-dependent field. The result of this averaging is that magnetic fields with frequencies below ~1 MHz should have much smaller effects than a static field of comparable strength. For example, in the presence of the Earth’s magnetic field (~50 μT), a 1 µT-strength, 50 or 60 Hz magnetic field is predicted to have a similar effect to that of a ~10 nT change in the Earth’s field (58). The latter would be experienced by travelling 2.5 km towards or away from the geomagnetic north pole (58). Similar conclusions apply to any frequency up to ~100 kHz. Stronger time-dependent magnetic fields are expected to lead to larger effects but still smaller than for a static field of similar strength.

## High-frequency magnetic field effects (> 1 MHz)

The situation is different for magnetic fields that vary during the lifetime of a radical pair, i.e. for frequencies above ~1 MHz. Magnetic field effects can be expected if the oscillation frequency is in resonance with an S↔T interconversion frequency or, equivalently, if it matches the energy gap between two of the spin energy-levels of the radicals (60–63). Unless the time-dependent field is very strong, non-resonant effects are extremely weak.

In a static magnetic field stronger than the hyperfine, exchange and dipolar interactions, the dominant resonance frequency can be calculated using the conversion factor of 28 MHz per mT, e.g. 2.8 GHz for a 100 mT static field (**Figures 1f, g**). This “Larmor-frequency” resonance is strongest when the static and time-dependent fields are perpendicular to one another and weakest when they are parallel. A magnetic field effect specifically at the Larmor frequency, with this dependence on the relative directions of the two fields, can be used as a diagnostic test for the operation of the radical pair mechanism (64).

By contrast, in weak static fields, comparable to or smaller than the internal magnetic interactions, a specific resonance at the Larmor frequency (e.g. 1.4 MHz for a 50 μT field) is not normally expected (14, 65). The only exception to this is when the exchange and dipolar interactions and the hyperfine interactions in one of the radicals are all extremely small (an unlikely event) (14, 66). For weak static fields, the maximum resonance frequency corresponds to the separation of the highest and lowest spin energy-levels (**Figure 1f**) (65), a prediction that has been used to guide behavioural experiments on the orientation of migratory songbirds exposed to radiofrequency magnetic fields (67–71). There may also be changes in reaction yields for frequencies comparable to the strengths of the internal magnetic interactions, e.g. ~28 MHz for ~1 mT hyperfine couplings.

## Magnetic isotope effects

Isotopic substitution changes the spin and magnetic moment of a nucleus, and therefore its hyperfine interaction, leading to a “magnetic isotope effect” (62, 72–74) quite distinct from the more familiar mass, or kinetic, isotope effect. Like external magnetic fields, isotopic substitution can increase or decrease reaction yields depending on the initial spin state of the radical pair and the spin state of the product. Other things being equal, the largest magnetic isotope effects are expected when a non-magnetic nuclide is replaced by a magnetic one, or vice versa, for example ^12^C → ^13^C (75) or ^25^Mg → ^24^Mg and/or ^26^Mg (76–78). The latter has been suggested as a new way of selectively killing cancer cells (79, 80).

## Chemical effects

The discussion so far has considered exclusively “geminate” (G) radical pairs formed in spin-correlated S or T states from S or T precursors. This situation is very common, especially for photochemical reactions where the precursor is an electronically excited S or T molecule and the radicals are formed by electron or H-atom transfer or homolytic bond cleavage. However, there can also be “F-pairs”, arising from the random encounter, e.g. by diffusion in solution, of independently created radicals which initially have uncorrelated electron spins. The spin correlation required for magnetic field effects arises from subsequent spin-selective reactions. F-pairs behave qualitatively like triplet G-pairs if the S state reacts faster than the T state, and like singlet G-pairs if the opposite is true (81, 82). The most common case is that S-pairs react to form stable singlet-state products while T-pairs are unreactive. Kinetic competition between the spin-selective reaction of the S-pair and diffusive separation of both S- and T-pairs [**Figure 1d**(i)] gives rise to the magnetic sensitivity.

## Amplification mechanisms

There has been a number of suggestions of mechanisms that could amplify small magnetic field effects [briefly reviewed in (58)]. One proposal is that a paramagnetic scavenger, reacting with one of the constituents of a radical pair, could not only boost its sensitivity to magnetic fields but also circumvent the detrimental effects of rapid spin relaxation (83–86). Such a mechanism might allow superoxide-containing radical pairs (see below) to be magnetically sensitive.

A second possibility is that the chemical feedback and autocatalysis that are features of oscillating chemical reactions could permit small magnetically-induced changes in the kinetics of radical pair intermediates to have a disproportionate effect on the amplitude of the oscillations (87–94). Interestingly, it has been proposed that related effects could arise in an intrinsically oscillatory system of coupled mitochondria in cancer cells (95).

## Concluding remarks

Magnetobiology has a vast literature, much of it beset by conflicting, implausible or extravagant claims (11). That so many reports of non-thermal biological magnetic field effects have been attributed to the radical pair mechanism seems to owe more to the scarcity of plausible alternatives than to solid experimental evidence of radical pair biochemistry. Assignment of a magnetic field effect to the radical pair mechanism is generally more convincing if the experimental observations do not conflict with theoretical predictions.

The theoretical basis of the radical pair mechanism has become well-established over the last 50 years, to the extent that upper limits on the magnetic sensitivity of radical pair reactions can be estimated quite reliably if enough is known, or can be inferred, about the properties of the radicals involved (58, 71, 96–99). Such calculations can help one decide whether an observed effect is likely to have a radical-pair origin or, sometimes, whether it is likely to be an experimental artefact.

An example of the utility of spin dynamics calculations is provided by the (independently replicated) finding that migratory birds are prevented from orienting in the Earth’s magnetic field (~50 µT) when exposed to astonishingly weak (~1-100 nT) radiofrequency (~1-100 MHz) magnetic fields (66–71, 100–103). Although evidence is accumulating in support of the notion that light-induced radical pairs (formed in cryptochrome proteins located in photoreceptor cells in the birds’ retinas) could form the basis of the avian magnetic compass sensor, it is still unclear whether they are sensitive enough to allow small nocturnal migrants to derive a compass bearing with only starlight available to initiate the radical-pair chemistry. It is much more a stretch of the imagination to believe that this sensory mechanism could be disrupted by time-dependent magnetic fields ~1000 times weaker than the geomagnetic field. Computer simulations of realistic spin-systems are being used to guide behavioural experiments by predicting which radiofrequencies should and which should not cause the birds to be disoriented (65, 71). The hope is that such a combination of theory and experiment will reveal whether radiofrequency disorientation is an informative side effect, an experimental artefact or, conceivably, a biologically relevant phenomenon.

Finally, a brief comment on reactive oxygen species (ROS, e.g. ${\text{O}}_{2}^{•-}$ and ${\text{OH}}^{•}$ ) and reactive nitrogen species (e.g. ${\text{NO}}^{•}$ ), some of which play crucial roles in cell signalling and oxidative damage. Various ROS-related effects of static, time-dependent and even hypomagnetic (i.e. << 50 μT) fields, have been discussed in the context of the radical pair mechanism, with the emphasis on superoxide ( ${\text{O}}_{2}^{•-}$ ) (15, 20, 104–115). A property shared by ${\text{O}}_{2}^{•-}$ , ${\text{OH}}^{•}$ , and ${\text{NO}}^{•}$ is that they all spin-relax much more rapidly (nanoseconds or faster) than the vast majority of organic radicals (116, 117). As a consequence, extremely small magnetic field effects can be expected for radical pairs containing these radicals, even for very strong fields (118, 119). Any radical-pair effects on ROS levels in living systems are much more likely to arise from upstream pairs of slower-relaxing organic radicals (11).

## References

[1] Adair RK. Effects of very weak magnetic fields on radical pair reformation. Bioelectromagnetics. (1999) 20:255–63. doi: 10.1002/(SICI)1521-186X(1999)20:4<255::AID-BEM6>3.0.CO;2-W 10230939

[2] Swanson J Kheifets L. Biophysical mechanisms: a component in the weight of evidence for health effects of power-frequency electric and magnetic fields. Radiat Res. (2006) 165:470–8. doi: 10.1667/RR3522.1 16579660

[3] Steiner UE Ulrich T. Magnetic field effects in chemical kinetics and related phenomena. Chem Rev. (1989) 89:51–147. doi: 10.1021/cr00091a003

[4] McLauchlan KA Steiner UE. The spin-correlated radical pair as a reaction intermediate. Mol Phys. (1991) 73:241–63. doi: 10.1080/00268979100101181

[5] Scaiano JC Cozens FL Mclean J. Model for the rationalization of magnetic field effects in *vivo.* Application of the radical pair mechanism to biological systems. Photochem Photobiol. (1994) 59:585–9. doi: 10.1111/j.1751-1097.1994.tb09660.x 8066117

[6] Brocklehurst B McLauchlan KA. Free radical mechanism for the effects of environmental electromagnetic fields on biological systems. Int J Radiat Biol. (1996) 69:3–24. doi: 10.1080/095530096146147 8601753

[7] Brocklehurst B. Magnetic fields and radical reactions: recent developments and their role in Nature. Chem Soc Rev. (2002) 31:301–11. doi: 10.1039/b107250c 12357727

[8] Woodward JR. Radical pairs in solution. Prog React Kinet Mech. (2002) 27:165–207. doi: 10.3184/007967402103165388

[9] Rodgers CT. Magnetic field effects in chemical systems. Pure Appl Chem. (2009) 81:19–43. doi: 10.1351/PAC-CON-08-10-18

[10] Jones AR. Magnetic field effects in proteins. Mol Phys. (2016) 114:1691–702. doi: 10.1080/00268976.2016.1149631

[11] Hore PJ. Spin chemistry in living systems. Natl Sci Rev. (2024) 11:nwae126. doi: 10.1093/nsr/nwae126 39144744 PMC11321246

[12] Schulten K Swenberg CE Weller A. A biomagnetic sensory mechanism based on magnetic field modulated coherent electron spin motion. Z Phys Chem NF. (1978) 111:1–5. doi: 10.1524/zpch.1978.111.1.001

[13] Ritz T Adem S Schulten K. A model for photoreceptor-based magnetoreception in birds. Biophys J. (2000) 78:707–18. doi: 10.1016/S0006-3495(00)76629-X PMC1300674 10653784

[14] Hore PJ Mouritsen H. The radical pair mechanism of magnetoreception. Annu Rev Biophys. (2016) 45:299–344. doi: 10.1146/annurev-biophys-032116-094545 27216936

[15] Nordmann GC Hochstoeger T Keays DA. Magnetoreception - a sense without a receptor. PloS Biol. (2017) 15:e2003234. doi: 10.1371/journal.pbio.2003234 29059181 PMC5695626

[16] Mouritsen H. Long-distance navigation and magnetoreception in migratory animals. Nature. (2018) 558:50–9. doi: 10.1038/s41586-018-0176-1 29875486

[17] Johnsen S Lohmann KJ Warrant EJ. Animal navigation: a noisy magnetic sense? J Exp Biol. (2020) 223:jeb164921. doi: 10.1242/jeb.164921 32967977

[18] Binhi VN Prato FS. Biological effects of the hypomagnetic field: an analytical review of experiments and theories. PloS One. (2017) 12:e0179340. doi: 10.1371/journal.pone.0179340 28654641 PMC5487043

[19] Zhang XL Yarema K Xu A. Biological effects of static magnetic fields. Singapore: Springer (2017).

[20] Pooam M El-Esawi M Aguida B Ahmad M. Cryptochrome and quantum biology: new insights for plant science and crop improvement. J Plant Biochem Biotech. (2020) 29:636–51. doi: 10.1007/s13562-020-00620-6

[21] Buchachenko AL Bukhvostov AA Ermakov KV Kuznetsov DA. A specific role of magnetic isotopes in biological and ecological systems. Physics and biophysics beyond. Prog Biophys Mol Biol. (2020) 155:1–19. doi: 10.1016/j.pbiomolbio.2020.02.007 32224188

[22] Kim Y Bertagna F D’Souza EM Heyes DJ Johannissen LO Nery ET. Quantum biology: An update and perspective. Quantum Rep. (2021) 3:1–48. doi: 10.3390/quantum3010006

[23] Binhi VN Rubin AB. Theoretical concepts in magnetobiology after 40 years of research. Cells. (2022) 11:274. doi: 10.3390/cells11020274 35053390 PMC8773520

[24] Zadeh-Haghighi H Simon C. Magnetic field effects in biology from the perspective of the radical pair mechanism. J R Soc Interface. (2022) 19:20220325. doi: 10.1098/rsif.2022.0325 35919980 PMC9346374

[25] Ueno S Shigemitsu T. Bioelectromagnetism. History, foundations and applications. Boca Raton: CRC Press (2022).

[26] Koltover VK. Magnetic isotope effects and nuclear spin catalysis in living cells and biomolecular motors: recent advances and future outlooks. Biophys Rev. (2023) 15:999–1006. doi: 10.1007/s12551-023-01162-6 37974974 PMC10643427

[27] Sarimov RM Serov DA Gudkov SV. Biological effects of magnetic storms and ELF magnetic fields. Biology. (2023) 12:1506. doi: 10.3390/biology12121506 38132332 PMC10740910

[28] Sarimov RM Serov DA Gudkov SV. Hypomagnetic conditions and their biological action. Biology. (2023) 12:1513. doi: 10.3390/biology12121513 38132339 PMC10740674

[29] Buchachenko AL. Magnetic effects across biochemistry, molecular biology and environmental chemistry. London: Academic Press (2024).

[30] Tota M Jonderko L Witek J Novickij V Kulbacka J. Cellular and molecular effects of magnetic fields. Int J Mol Sci. (2024) 25:8973. doi: 10.3390/ijms25168973 39201657 PMC11354277

[31] Timmel CR Till U Brocklehurst B McLauchlan KA Hore PJ. Effects of weak magnetic fields on free radical recombination reactions. Mol Phys. (1998) 95:71–89. doi: 10.1080/00268979809483134 11098854

[32] Hayashi H. Introduction to Dynamic Spin Chemistry. Singapore: World Scientific Publisher (2004).

[33] Uhlenbeck GE Goudsmit S. Ersetzung der Hypothese vom unmechanischen Zwang durch eine Forderung bezüglich des inneren Verhaltens jedes einzelnen Elektrons. Naturwissenschaften. (1925) 13:953–4.

[34] Tomonaga S. The Story of Spin. Chicago: University of Chicago Press (1997).

[35] Kuprov I. Spin. Cham, Switzerland: Springer (2023).

[36] Mims D Herpich J Lukzen NN Steiner UE Lambert C. Readout of spin quantum beats in a charge-separated radical pair by pump-push spectroscopy. Science. (2021) 374:1470–4. doi: 10.1126/science.abl4254 34914495

[37] Gilch P Pollinger-Dammer F Musewald C Michel-Beyerle ME Steiner UE. Magnetic field effect on picosecond electron transfer. Science. (1998) 281:982–4. doi: 10.1126/science.281.5379.982 9703512

[38] Bagryansky VA Borovkov VI Molin YN. Quantum beats in radical pairs. Russ Chem Rev. (2007) 76:493–506. doi: 10.1070/RC2007v076n06ABEH003715

[39] Rodgers CT Hore PJ. Chemical magnetoreception in birds: a radical pair mechanism. Proc Natl Acad Sci USA. (2009) 106:353–60. doi: 10.1073/pnas.0711968106 PMC2626707 19129499

[40] Kattnig DR Solov’yov IA Hore PJ. Electron spin relaxation in cryptochrome-based magnetoreception. Phys Chem Chem Phys. (2016) 18:12443–56. doi: 10.1039/C5CP06731F 27020113

[41] Weller A Nolting F Staerk H. A quantitative interpretation of the magnetic-field effect on hyperfine-coupling-induced triplet formation from radical ion-pairs. Chem Phys Lett. (1983) 96:24–7. doi: 10.1016/0009-2614(83)80109-2

[42] Michel-Beyerle ME Haberkorn R Bube W Steffens E Schröder H Neusser HJ. Magnetic-field modulation of geminate recombination of radical ions in a polar solvent. Chem Phys. (1976) 17:139–45. doi: 10.1016/0301-0104(76)80097-3

[43] Michel-Beyerle ME Krüger HW Haberkorn R Seidlitz H. Nanosecond time-resolved magnetic field effect on radical recombination in solution. Chem Phys. (1979) 42:441–7. doi: 10.1016/0301-0104(79)80094-4

[44] Werner HJ Schulten K Weller A. Electron-transfer and spin exchange contributing to magnetic-field dependence of primary photo-chemical reaction of bacterial photosynthesis. Biochim Biophys Acta. (1978) 502:255–68. doi: 10.1016/0005-2728(78)90047-6 306834

[45] Wong SY Benjamin P Hore PJ. Magnetic field effects on radical pair reactions: estimation of *B* _1/2_ for flavin-tryptophan radical pairs in cryptochromes. Phys Chem Chem Phys. (2023) 25:975–82. doi: 10.1039/D2CP03793A PMC9811481 36519379

[46] Golesworthy M Zollitsch T Luo J Selby D Jarocha LE Henbest KB. Singlet-triplet dephasing in radical pairs in avian cryptochromes leads to time-dependent magnetic field effects. J Chem Phys. (2023) 159:105102. doi: 10.1063/5.0166675 37694754

[47] Maeda K Robinson AJ Henbest KB Hogben HJ Biskup T Ahmad M. Magnetically sensitive light-induced reactions in cryptochrome are consistent with its proposed role as a magnetoreceptor. Proc Natl Acad Sci USA. (2012) 109:4774–9. doi: 10.1073/pnas.1118959109 PMC3323948 22421133

[48] Kim T Kim J Ke XS Brewster JT Oh J Sessler JL. Magnetic-field-induced modulation of charge-recombination dynamics in a rosarin-fullerene complex. Angew Chem Int Ed. (2021) 60:9379–83. doi: 10.1002/anie.202017332 33590640

[49] Player TC Hore PJ. Source of magnetic field effects on the electrocatalytic reduction of CO_2_ . J Chem Phys. (2020) 153:084303. doi: 10.1063/5.0021643 32872863

[50] Hayashi H Nagakura S. Theoretical study of relaxation mechanism in magnetic-field effects on chemical-reactions. Bull Chem Soc Jpn. (1984) 57:322–8. doi: 10.1246/bcsj.57.322

[51] Cintolesi F Ritz T Kay CWM Timmel CR Hore PJ. Anisotropic recombination of an immobilized photoinduced radical pair in a 50-μT magnetic field: a model avian photomagnetoreceptor. Chem Phys. (2003) 294:385–99. doi: 10.1016/S0301-0104(03)00320-3

[52] Maeda K Henbest KB Cintolesi F Kuprov I Rodgers CT Liddell PA. Chemical compass model of avian magnetoreception. Nature. (2008) 453:387–90. doi: 10.1038/nature06834 18449197

[53] Kerpal C Richert S Storey JG Pillai S Liddell PA Gust D. Chemical compass behaviour at microtesla magnetic fields strengthens the radical pair hypothesis of avian magnetoreception. Nat Commun. (2019) 10:3707. doi: 10.1038/s41467-019-11655-2 31420558 PMC6697675

[54] Lewis A. Spin Dynamics in Radical Pairs. Cham, Switzerland: Springer International Publishing (2018).

[55] Luo J Hore PJ. Chiral-induced spin selectivity in the formation and recombination of radical pairs: cryptochrome magnetoreception and EPR detection. New J Phys. (2021) 23:043032. doi: 10.1088/1367-2630/abed0b

[56] Weiss EA Ratner MA Wasielewski MR. Direct measurement of singlet-triplet splitting within rodlike photogenerated radical ion pairs using magnetic field effects: estimation of the electronic coupling for charge recombination. J Phys Chem A. (2003) 107:3639–47. doi: 10.1021/jp0224315

[57] Efimova O Hore PJ. Role of exchange and dipolar interactions in the radical pair model of the avian magnetic compass. Biophys J. (2008) 94:1565–74. doi: 10.1529/biophysj.107.119362 PMC2242753 17981903

[58] Hore PJ. Upper bound on the biological effects of 50/60 Hz magnetic fields mediated by radical pairs. eLife. (2019) 8:e44179. doi: 10.7554/eLife.44179.016 30801245 PMC6417859

[59] Scaiano JC Mohtat N Cozens FL Mclean J Thansandote A. Application of the radical pair mechanism to free radicals in organized systems: can the effects of 60 Hz be predicted from studies under static fields? Bioelectromagnetics. (1994) 15:549–54. doi: 10.1002/bem.2250150608 7880168

[60] Bowman MK Budil DE Closs GL Kostka AG Wraight CA Norris JR. Magnetic-resonance spectroscopy of the primary state, P^F^, of bacterial photosynthesis. Proc Natl Acad Sci USA. (1981) 78:3305–7. doi: 10.1073/pnas.78.6.3305 PMC319556 16593028

[61] Lersch W Michel-Beyerle ME. RYDMR - theory and applications. In: Hoff AJ, editor. Advanced EPR. Applications in biology and biochemistry. Elsevier, Amsterdam (1989). p. 685–705.

[62] Woodward JR Timmel CR McLauchlan KA Hore PJ. Radio frequency magnetic field effects on electron-hole recombination. Phys Rev Lett. (2001) 87:077602. doi: 10.1103/PhysRevLett.87.077602 11497916

[63] Luo J Benjamin P Gerhards L Hogben HJ Hore PJ. Orientation of birds in radiofrequency fields in the absence of the Earth’s magnetic field: a possible test for the radical pair mechanism of magnetoreception. J R Soc Interface. (2024) 21:20240133. doi: 10.1098/rsif.2024.0133 39110232 PMC11305414

[64] Henbest KB Kukura P Rodgers CT Hore PJ Timmel CR. Radio frequency magnetic field effects on a radical recombination reaction: a diagnostic test for the radical pair mechanism. J Amer Chem Soc. (2004) 126:8102–3. doi: 10.1021/ja048220q 15225036

[65] Hiscock HG Mouritsen H Manolopoulos DE Hore PJ. Disruption of magnetic compass orientation in migratory birds by radiofrequency electromagnetic fields. Biophys J. (2017) 113:1475–84. doi: 10.1016/j.bpj.2017.07.031 PMC5627152 28978441

[66] Ritz T Wiltschko R Hore PJ Rodgers CT Stapput K Thalau P. Magnetic compass of birds is based on a molecule with optimal directional sensitivity. Biophys J. (2009) 96:3451–7. doi: 10.1016/j.bpj.2008.11.072 PMC2718301 19383488

[67] Engels S Schneider NL Lefeldt N Hein CM Zapka M Michalik A. Anthropogenic electromagnetic noise disrupts magnetic compass orientation in a migratory bird. Nature. (2014) 509:353–6. doi: 10.1038/nature13290 24805233

[68] Schwarze S Schneider N-L Reichl T Dreyer D Lefeldt N Engels S. Weak broadband electromagnetic fields are more disruptive to magnetic compass orientation in a night-migratory songbird (*Erithacus rubecula*) than strong narrow-band fields. Front Behav Neurosci. (2016) 10:55. doi: 10.3389/fnbeh.2016.00055 27047356 PMC4801848

[69] Kobylkov D Wynn J Winklhofer M Chetverikova R Xu JJ Hiscock H. Electromagnetic 0.1-100 kHz noise does not disrupt orientation in a night-migrating songbird implying a spin coherence lifetime of less than 10 microseconds. J R Soc Interface. (2019) 16:20190716. doi: 10.1098/rsif.2019.0716 31847760 PMC6936046

[70] Leberecht B Kobylkov D Karwinkel T Doge S Burnus L Wong SY. Broadband 75-85 MHz radiofrequency fields disrupt magnetic compass orientation in night-migratory songbirds consistent with a flavin-based radical pair magnetoreceptor. J Comp Physiol A. (2022) 208:97–106. doi: 10.1007/s00359-021-01537-8 PMC8918455 35019998

[71] Leberecht B Wong SY Satish B Döge S Hindman J Venkatraman L. Upper bound for broadband radiofrequency field disruption of magnetic compass orientation in night-migratory songbirds. Proc Natl Acad Sci USA. (2023) 120:2301153120. doi: 10.1073/pnas.2301153120 PMC10334787 37399422

[72] Salikhov KM. Magnetic isotope effect in radical reactions. Vienna: Springer-Verlag (1996).

[73] Rodgers CT Norman SA Henbest KB Timmel CR Hore PJ. Determination of radical re-encounter probability distributions from magnetic field effects on reaction yields. J Amer Chem Soc. (2007) 129:6746–55. doi: 10.1021/ja068209l 17469816

[74] Buchachenko AL. Magnetic isotope effect in chemistry and biochemistry. New York: Nova Science Publishers (2009).

[75] Pazera GJ Benjamin P Mouritsen H Hore PJ. Isotope substitution effects on the magnetic compass properties of cryptochrome-based radical pairs: a computational study. J Phys Chem B. (2023) 127:838–45. doi: 10.1021/acs.jpcb.2c05335 PMC9900586 36669149

[76] Buchachenko AL Kouznetsov DA Orlova MA Markarian AA. Magnetic isotope effect of magnesium in phosphoglycerate kinase phosphorylation. Proc Natl Acad Sci USA. (2005) 102:10793–6. doi: 10.1073/pnas.0504876102 PMC1182455 16043694

[77] Buchachenko AL Kouznetsov DA Breslavskaya NN Orlova MA. Magnesium isotope effects in enzymatic phosphorylation. J Phys Chem B. (2008) 112:2548–56. doi: 10.1021/jp710989d 18247604

[78] Stovbun SV Zlenko DV Bukhvostov AA Vedenkin AS Skoblin AA Kuznetsov DA. Magnetic field and nuclear spin influence on the DNA synthesis rate. Sci Rep. (2023) 13:465. doi: 10.1038/s41598-022-26744-4 36627313 PMC9832033

[79] Buchachenko AL Kuznetsov DA. Genes and cancer under magnetic control. Russ J Phys Chem B. (2021) 15:1–11. doi: 10.1134/S1990793121010024

[80] Buchachenko A Bukhvostov A Ermakov K Kuznetsov D. Nuclear spin selectivity in enzymatic catalysis: A caution for applied biophysics. Arch Biochem Biophys. (2019) 667:30–5. doi: 10.1016/j.abb.2019.04.005 31029686

[81] Muus LT Atkins PW McLauchlan KA Pedersen JB. Chemically induced magnetic polarization. Dordrecht: D. Reidel (1977).

[82] Salikhov KM Molin YN Sagdeev RZ Buchachenko AL. Spin Polarization and Magnetic Field Effects in Radical Reactions. New York: Elsevier (1984).

[83] Kattnig DR Hore PJ. The sensitivity of a radical pair compass magnetoreceptor can be significantly amplified by radical scavengers. Sci Rep. (2017) 7:11640. doi: 10.1038/s41598-017-09914-7 28912470 PMC5599710

[84] Kattnig DR. Radical-pair-based magnetoreception amplified by radical scavenging: resilience to spin relaxation. J Phys Chem B. (2017) 121:10215–27. doi: 10.1021/acs.jpcb.7b07672 29028342

[85] Babcock NS Kattnig DR. Radical scavenging could answer the challenge posed by electron-electron dipolar interactions in the cryptochrome compass model. Jacs Au. (2021) 1:2033–46. doi: 10.1021/jacsau.1c00332 PMC8611662 34841416

[86] Deviers J Cailliez F de la Lande A Kattnig DR. Anisotropic magnetic field effects in the re-oxidation of cryptochrome in the presence of scavenger radicals. J Chem Phys. (2022) 156:025101. doi: 10.1063/5.0078115 35032990

[87] Eichwald C Walleczek J. Model for magnetic field effects on radical pair recombination in enzyme kinetics. Biophys J. (1996) 71:623–31. doi: 10.1016/S0006-3495(96)79263-9 PMC1233520 8842202

[88] Eichwald C Walleczek J. Low-frequency-dependent effects of oscillating magnetic fields on radical pair recombination in enzyme kinetics. J Chem Phys. (1997) 107:4943–50. doi: 10.1063/1.474858

[89] Eichwald C Walleczek J. Magnetic field perturbations as a tool for controlling enzyme-regulated and oscillatory biochemical reactions. Biophys Chem. (1998) 74:209–24. doi: 10.1016/S0301-4622(98)00180-X 17029747

[90] Møller AC Olsen LF. Effect of magnetic fields on an oscillating enzyme reaction. J Am Chem Soc. (1999) 121:6351–4. doi: 10.1021/ja990834l

[91] Møller AC Lunding A Olsen LF. Further studies of the effect of magnetic fields on the oscillating peroxidase-oxidase reaction. Phys Chem Chem Phys. (2000) 2:3443–6. doi: 10.1039/b003641m

[92] Møller AC Olsen LF. Perturbations of simple oscillations and complex dynamics in the peroxidase-oxidase reaction using magnetic fields. J Phys Chem B. (2000) 104:140–6. doi: 10.1021/jp993284m

[93] Purtov PA. External magnetic fields as a possible cause of stability disturbance of stationary states far from equilibrium in reactions involving radical pairs. Appl Magn Reson. (2004) 26:83–97. doi: 10.1007/BF03166564

[94] Player TC Baxter EDA Allatt S Hore PJ. Amplification of weak magnetic field effects on oscillating reactions. Sci Rep. (2021) 11:9615. doi: 10.1038/s41598-021-88871-8 33953230 PMC8100163

[95] Zandieh A Shariatpanahi SP Ravassipour AA Azadipour J Nezamtaheri MS Habibi-Kelishomi Z. An amplification mechanism for weak ELF magnetic fields quantum-bio effects in cancer cells. Sci Rep. (2025) 15:2964. doi: 10.1038/s41598-025-87235-w 39849096 PMC11757740

[96] Hiscock HG Hiscock TW Kattnig DR Scrivener T Lewis AM Manolopoulos DE. Navigating at night: fundamental limits on the sensitivity of radical pair magnetoreception under dim light. Q Rev Biophys. (2019) 52:e9. doi: 10.1017/S0033583519000076 31637984

[97] Ren Y Hiscock H Hore PJ. Angular precision of radical pair compass magnetoreceptors. Biophys J. (2021) 120:547–55. doi: 10.1016/j.bpj.2020.12.023 PMC7896030 33421412

[98] Gerhards L Nielsen C Kattnig DR Hore PJ Solov’yov IA. Modeling spin relaxation in complex radical systems using. MolSpin J Comput Chem. (2023) 44:1704–14. doi: 10.1002/jcc.v44.19 37186467

[99] Pazera GJ Fay TP Solov’yov IA Hore PJ Gerhards L. Spin dynamics of radical pairs using the stochastic Schrödinger equation in. MolSpin J Chem Theory Comput. (2024) 20:8412–21. doi: 10.1021/acs.jctc.4c00361 PMC11465467 39283312

[100] Ritz T Thalau P Phillips JB Wiltschko R Wiltschko W. Resonance effects indicate a radical-pair mechanism for avian magnetic compass. Nature. (2004) 429:177–80. doi: 10.1038/nature02534 15141211

[101] Thalau P Ritz T Stapput K Wiltschko R Wiltschko W. Magnetic compass orientation of migratory birds in the presence of a 1.315 MHz oscillating field. Naturwissenschaften. (2005) 92:86–90. doi: 10.1007/s00114-004-0595-8 15614508

[102] Kavokin K Chernetsov N Pakhomov A Bojarinova J Kobylkov D Namozov B. Magnetic orientation of garden warblers (*Sylvia borin*) under 1.4 MHz radiofrequency magnetic field. J R Soc Interface. (2014) 11:20140451. doi: 10.1098/rsif.2014.0451 24942848 PMC4208380

[103] Pakhomov A Bojarinova J Cherbunin R Chetverikova R Grigoryev PS Kavokin K. Very weak oscillating magnetic field disrupts the magnetic compass of songbird migrants. J R Soc Interface. (2017) 14:20170364. doi: 10.1098/rsif.2017.0364 28794163 PMC5582129

[104] Usselman RJ Hill I Singel DJ Martino CF. Spin biochemistry modulates reactive oxygen species (ROS) production by radio frequency magnetic fields. PloS One. (2014) 9:e93065. doi: 10.1371/journal.pone.0093065 24681944 PMC3969378

[105] Usselman RJ Chavarriaga C Castello PR Procopio M Ritz T Dratz EA. The quantum biology of reactive oxygen species partitioning impacts cellular bioenergetics. Sci Rep. (2016) 6:38543. doi: 10.1038/srep38543 27995996 PMC5172244

[106] Sherrard RM Morellini N Jourdan N El-Esawi M Arthaut LD Niessner C. Low-intensity electromagnetic fields induce human cryptochrome to modulate intracellular reactive oxygen species. PloS Biol. (2018) 16:e2006229. doi: 10.1371/journal.pbio.2006229 30278045 PMC6168118

[107] Juutilainen J Herrala M Luukkonen J Naarala J Hore PJ. Magnetocarcinogenesis: is there a mechanism for carcinogenic effects of weak magnetic fields? Proc R Soc B. (2018) 285:20180590. doi: 10.1098/rspb.2018.0590 PMC5998098 29794049

[108] Lai H. Exposure to static and extremely-low frequency electromagnetic fields and cellular free radicals. Electromagn Biol Med. (2019) 38:231–48. doi: 10.1080/15368378.2019.1656645 31450976

[109] Pooam M Jourdan N El Esawi M Sherrard RM Ahmad M. HEK293 cell response to static magnetic fields via the radical pair mechanism may explain therapeutic effects of pulsed electromagnetic fields. PloS One. (2020) 15:e0243038. doi: 10.1371/journal.pone.0243038 33270696 PMC7714230

[110] Tofani S. Magnetic fields and apoptosis: a possible mechanism. Electromagn Biol Med. (2022) 41:293–303. doi: 10.1080/15368378.2022.2073547 35543158

[111] Rishabh R Zadeh-Haghighi H Salahub D Simon C. Radical pairs may explain reactive oxygen species-mediated effects of hypomagnetic field on neurogenesis. PloS Comput Biol. (2022) 18:e1010198. doi: 10.1371/journal.pcbi.1010198 35653379 PMC9197044

[112] Thöni V Mauracher D Ramalingam A Fiechtner B Sandbichler AM Egg M. Quantum based effects of therapeutic nuclear magnetic resonance persistently reduce glycolysis. Iscience. (2022) 25:105536. doi: 10.1016/j.isci.2022.105536 36444297 PMC9700021

[113] Thöni V Dimova EY Kietzmann T Usselman RJ Egg M. Therapeutic nuclear magnetic resonance and intermittent hypoxia trigger time dependent on/off effects in circadian clocks and confirm a central role of superoxide in cellular magnetic field effects. Redox Biol. (2024) 72:103152. doi: 10.1016/j.redox.2024.103152 38593630 PMC11016797

[114] Kinsey LJ Van Huizen AV Beane WS. Weak magnetic fields modulate superoxide to control planarian regeneration. Front Phys. (2023) 10:1086809. doi: 10.3389/fphy.2022.1086809

[115] Austvold CK Keable SM Procopio M Usselman RJ. Quantitative measurements of reactive oxygen species partitioning in electron transfer flavoenzyme magnetic field sensing. Front Physiol. (2024) 15:1348395. doi: 10.3389/fphys.2024.1348395 38370016 PMC10869518

[116] Hogben HJ Efimova O Wagner-Rundell N Timmel CR Hore PJ. Possible involvement of superoxide and dioxygen with cryptochrome in avian magnetoreception: origin of Zeeman resonances observed by *in vivo* EPR spectroscopy. Chem Phys Lett. (2009) 480:118–22. doi: 10.1016/j.cplett.2009.08.051

[117] Player TC Hore PJ. Viability of superoxide-containing radical pairs as magnetoreceptors. J Chem Phys. (2019) 151:225101. doi: 10.1063/1.5129608 31837685

[118] Karogodina TY Sergeeva SV Stass DV. Magnetic field effect in the reaction of recombination of nitric oxide and superoxide anion. Appl Magn Reson. (2009) 36:195–208. doi: 10.1007/s00723-009-0018-2

[119] Karogodina TY Dranov IG Sergeeva SV Stass DV Steiner UE. Kinetic magnetic-field effect involving the small biologically relevant inorganic radicals nitric oxide and superoxide. Chem Phys Chem. (2011) 12:1714–28. doi: 10.1002/cphc.201100178 21598373

