# Supplementary material for: Magneto-oncology: a radical pair primer
Source: Front Oncol. 2025 Mar 7;15:1539718. doi: 10.3389/fonc.2025.1539718 (PMC11925880; doi:10.3389/fonc.2025.1539718)
Supplement: Supplementary file 1 [file Presentation1.pdf]

Magneto-oncology: a radical pair primer

P. J. Hore

Department of Chemistry, Oxford University, Oxford, UK
peter.hore@chem.ox.ac.uk

Supplementary material

Constants

γ_e	1.76×10^{11} T^{-1} s^{-1}	Gyromagnetic ratio of the electron
h	6.626×10^{-34} J s	Planck's constant
k_B	1.381×10^{-23} J K^{-1}	Boltzmann's constant
μ_B	9.274×10^{-24} J T^{-1}	Bohr magneton

- The energy of the Zeeman interaction of a radical in a static magnetic field B_0 is $\Delta E_Z = h\nu_L = g\mu_B B_0$ where ν_L is the electron Larmor frequency and g is the g-value of the radical. For a free electron, $g = g_e = 2.002319$. The g-values of most organic radicals differ from g_e by no more than ~1%.

- For a free electron, $\Delta E_Z = 1.86 \times 10^{-23} \times B_0$ and $\nu_L = 2.80 \times 10^{10} \times B_0$, with ΔE_Z in joules, ν_L in hertz (s^{-1}), and B_0 in teslas.

- At physiological temperature (T = 313 K), the Zeeman interaction, ΔE_Z, equals $k_B T$ when B_0 = 233 T. Typical hyperfine interactions are ~ 1 mT.

- The effective hyperfine interaction of a radical pair is given by:

$$a_{\text{eff}} = \sqrt{\frac{1}{3}\sum_j a_j^2 I_j\left(I_j+1\right)},$$

  in which a_j and I_j are the isotropic hyperfine coupling constant and the spin quantum number of nucleus j and the sum runs over all nuclei in the radical pair.

- The mid-point of the hyperfine magnetic field effect occurs at a static field $B_0 = B_{1/2}$ given by: $B_{1/2} \approx a_{\text{eff}}$.

- The mid-point of the Δg magnetic field effect occurs at a static field $B_0 = B_{1/2}^{(\Delta g)}$ given by:

$$B_{1/2}^{(\Delta g)} \approx \frac{h}{2\pi\, \Delta g\, \mu_B\, \tau},$$

  in which Δg is the difference in the g-values of the two radicals and τ is the lifetime of the spin coherence.

- A condition for the existence of a low field effect at a field B_0 is that the period of the electron Larmor precession, $\left(\gamma_e B_0 / 2\pi\right)^{-1}$, should not be much longer than the lifetime of the spin coherence.

- A condition for the existence of a resonant effect of a magnetic field with frequency > 1 MHz is that $\left(\gamma_{e} B_{1} / 2\pi\right)^{-1}$ should not be much longer than the lifetime of the spin coherence. B_1 is the strength of the time-dependent magnetic field.

- The resonance frequency for a pair of spin energy-levels separated by an energy ΔE is $\nu = \Delta E / h$.
